# Balancing between A and I

**DOI:** 10.2340/1651-226X.2025.43320

**Published:** 2025-05-07

**Authors:** Emma Skovgaard Pedersen, Christoffer Johansen, Mette Kielsholm Thomsen

**Affiliations:** a Department of Clinical Epidemiology, Aarhus University & Aarhus University Hospital, Aarhus, Denmark; b Danish Cancer Society National Cancer Survivorship and Late Effects Research Center (CASTLE), Department of Oncology, Copenhagen University Hospital - Rigshospitalet, Copenhagen Denmark

**Keywords:** Artificial intelligence, machine learning, precision medicine, diagnostic imaging, cancer therapy

Artificial intelligence (AI) in cancer is fast evolving, and the next step on the evolutional ladder is incorporating AI methods for risk stratification in precision oncology, but we need to call for balance between methodological rigor and clinical relevance. Currently, studies focus on either one, but rarely both aspects.

Ageing populations, increasing cancer incidence, and lack of health care personnel and resources [1] mean that technological assistance from AI applications are becoming ever more important, as potential tools to aid efficacy of diagnostic and treatment pathways, as well as prioritisation of services. This potential has been met by an increase in publications on AI in oncology; during the past two decades, the publication activity has moved from less than 10 papers per year up to more than a thousand papers annually, illustrating the interest in exploring the utility of these techniques in all aspects of cancer care (Figure 1).

**Figure 1:**
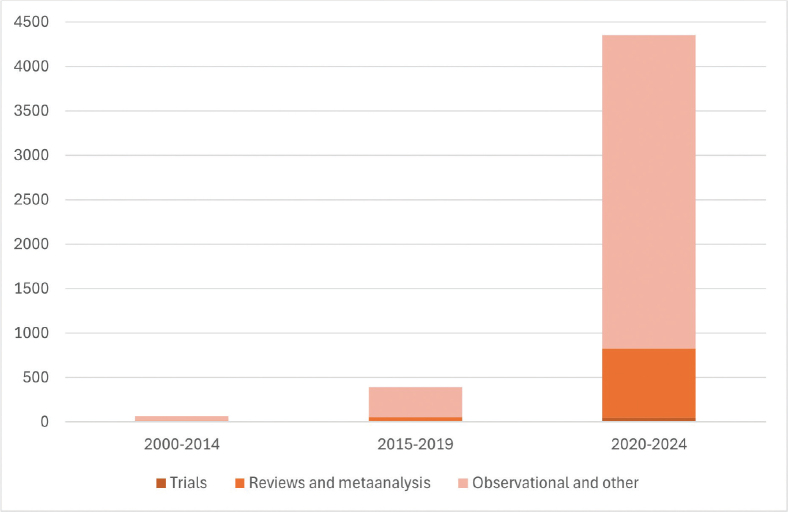
From 65 PubMed hits until 2014 to more than 4000 in 2020–2024: Reports with AI/ML and cancer in the title, by publication period and type of report.

AI areas of success currently span across cancer diagnosis, pre-treatment and treatment. AI tools have proven useful in aiding visual inspection of images in diagnosis and treatment of cancer patients, for example, during colonoscopies and for interpretation of mammogram images [2–4]. A review of clinical trials found that AI-tools increased adenoma detection during colonoscopies, with the increase driven by an improved detection of small adenomas, rather than advanced adenomas, underscoring that this tool can act as a supplement to colonoscopist expertise rather than a substitution [5]. In pre-treatment, an ongoing trial is investigating AI-assisted plans for perioperative care to optimise patients before surgery [6]. In treatment, AI tools may already be able to ease the workload of clinicians; an AI algorithm for contouring of scans prior to proton treatment of squamous cell carcinoma of the pharynx or larynx was shown to be more consistent within patients and were able to adhere more strictly to national guidelines than contours made by oncologists [7]. To plan and monitor treatment, AI methods are accelerating developments within circulating tumour DNA (ctDNA) for assessment of therapeutic response, by enhancing signal enrichment, making ctDNA monitoring applicable for personalised treatment strategies, and across more cancers than was previously possible [8].

While successful implementation of the above-mentioned AI tools into clinical practice constitutes a research area in itself [4, 9, 10], another major AI research area in cancer is developing high-performing prediction models using machine learning models, a subtype of AI [11]. Machine learning models have the potential to aid risk-stratification for screening, diagnosis, treatment planning, and prognostic prediction [12]. So far, this is mainly an academic pursuit, with a small minority of published prediction models achieving regulatory approval and subsequently being implemented into clinical practice [13]. Thus, the largest unharvested potential of AI in cancer, in our opinion, lies within prediction models for personalised oncology.

To enhance the use of AI prediction models in cancer research, we have identified two overarching sources of imbalance, that are currently tipping studies into the no-man’s-land between data science research and clinical medicine. These two challenges pertain to *expertise* and *alignment*.

## Expertise – in cancer *and* data science

Studies utilising machine learning for prediction models in cancer tend to suffer from one of two problems: First, studies performed by data scientists will often have a skilfully built model and include thorough documentation of the employed machine learning algorithms, but the models may not be clinically applicable or relevant, due to a lack of medical expertise, and results on actual model features and risk stratification utility are not sufficiently reported. A lack of epidemiological insight or understanding of the clinical reality may lead to issues such as conditioning on the future – for example, Zhang et al., included information on any second or third tumour to predict survival time from diagnoses of first tumour to death [14], resulting in inflated model performance. Another example is not acknowledging that in health care data, registrations are there for a reason, which means that healthy individuals are underrepresented in the development of AI tools [15, 16]. Medical records are a rich data source, but we should not blindly plunge into the vast amounts of data, without considering if the target population is represented by the data material.

Second, prediction studies led by clinicians may have a strong clinical foundation and relevance, but lack of technical and data expertise means that the resulting models may not be developed sufficiently to achieve adequate performance, and that reporting of methods is not sufficiently transparent [17]. These studies are at the same time especially prone to issues originating from lack of understanding of data structures and data management [18].

## Alignment – of purpose, results, and interpretation

A general theme in studies applying machine learning for prediction models in cancer is a lack of alignment between the purpose, results reported, and interpretation. When machine learning models are used for prediction, results tend to focus on metrics for model performance and comparison of various types of machine learning algorithms. Performance metrics are of course vital in judging which model is better [18], however, model performance by itself has limited clinical value. More transparency is needed in terms of which patient, tumour and treatment characteristics that lead to the prediction of low or high risk of the given outcome, or preferably the reporting of predictors included and a readily applicable model equation if possible and meaningful. Model output should be reported explicitly and in detail in the main text of papers reporting on machine learning models, rather than being relegated to supplementary materials [19]. We appreciate that explicit listing of features may wrongfully imply causal inference to some readers; thus, clear and precise wording of model specifications is required.

Fitting a prediction model is only one step on the road to utilising AI in clinical settings. However, it is not rare to see a study developing a prediction model and concluding that said model should promptly be implemented in practice. It can be helpful, as others have pointed out, to liken the process of AI development for health care with that of drug development [20]. In other words, the development of the specific prediction model (i.e. the phase 1 study) is followed by processes of evaluation, deployment, and monitoring of daily use (i.e. phase 2, 3 and 4), before the AI tool can be integrated into clinical settings. As such, all four phases are necessary to judge the clinical value, and we cannot go from phase 1 to concluding on the clinical utility of AI models. For this, pragmatic validation and pilot tests are necessary steps [21].

## The road ahead for AI in cancer research

To advance AI and machine learning in prediction studies within cancer, a road that is unlikely to be straightforward lies ahead. Researchers, healthcare engineers, and tech-savvy clinicians alike must largely pave the way as they go, in the absence of up-to-date, and interdisciplinary scientific guidelines. Technologies are evolving quicker than the usual pace of systematic overviews and collaborative efforts. The result of this situation is an abundance of publications and unimplemented models built by well-meaning researchers in a race against obsoleteness, wishing for a data-driven crystal ball to foretell the destiny of cancer patients [22]. No matter our optimism for technological advancements, it is crucial that we work together across disciplines to ensure the best possible balance between validity of models and interpretability and applicability of results.

## Data Availability

N/A.
